# Near infrared ray-guided surgery using Firefly technology of the daVinci Xi system and intraoperative upper gastrointestinal endoscopy for subtotal gastrectomy and surgery for cancer of the gastroesophageal junction

**DOI:** 10.1186/s12893-022-01633-9

**Published:** 2022-05-12

**Authors:** Hiroyuki Sagawa, Masaki Saito, Sunao Ito, Shunsuke Hayakawa, Shohei Ueno, Tomotaka Okubo, Tatsuya Tanaka, Ryo Ogawa, Hiroki Takahashi, Yoichi Matsuo, Akira Mitsui, Masahiro Kimura, Shuji Takiguchi

**Affiliations:** Department of Gastroenterological Surgery, Nagoya City University Graduate School of Medical Sciences, 1-Kawasumi, Mizuho-cho, Mizuho-ku, Nagoya, 467-8601 Japan

**Keywords:** Near infrared ray, Robotic surgery, Tumor marking, Gastric cancer, Adenocarcinoma of the gastroesophageal junction

## Abstract

**Background:**

In gastrectomies, especially subtotal gastrectomies and operations on the gastroesophageal junction, identifying the exact location of the tumor and establishing the appropriate resection line is very important. Accurate resection lines have a major impact on the preservation of organ function and curability. Preservation of as much as possible of the remaining stomach, including the fornix, may be an important surgical goal for maintaining an adequate postoperative quality of life. In adenocarcinoma of the gastroesophageal junction, the height of the esophageal dissection may affect reconstruction of the transhiatal approach.

**Methods:**

We perform a new technique, near infrared ray-guided surgery, for the accurate localization of a tumor using the Firefly technology of the daVinci Xi system and intra-operative upper gastrointestinal endoscopy. We used this new technique for cases of upper gastric cancer or adenocarcinoma of the gastroesophageal junction. In this retrospective study, we examined to determine the extent (mm) of the tumor invasion of the esophagus, visualization of near infrared ray contained within endoscopic light, and distance from the proximal margin of the tumor to the surgical cut line on rapid histopathology and in the permanent preparation, including the operative videos and extracted specimens.

**Results:**

We performed near infrared ray-guided surgery for 12 patients with gastric cancer or adenocarcinoma of the gastroesophageal junction, and the near infrared ray was clearly seen as green light with Firefly mode in all the patients. Near infrared ray-guided surgery was useful for obtaining localization of the tumor. In addition, it was possible to resect organ with adequate margins from tumor. Rapid intraoperative histopathological examinations confirmed that the resected specimens had negative margins. None of the patients required additional resection.

**Conclusions:**

We believe that because near infrared ray-guided surgery can provide an accurate resection line, it will be useful for the resection of upper gastric cancer and adenocarcinoma of the gastroesophageal junction. It will also provide patients with a good postoperative quality of life after surgery.

## Background

The surgical options for upper gastric cancer include total gastrectomy, proximal gastrectomy, and subtotal gastrectomy, which is a distal gastrectomy that preserves a small remnant stomach. Subtotal gastrectomy has been reported to be useful [1]. The standard therapy for upper gastric cancer is radical curative surgery. In addition, preservation of as much as possible of the remaining stomach, including the fornix, is an important surgical goal for providing a satisfactory postoperative quality of life (QOL). Ghrelin, which is an endogenous ligand of the growth hormone secretagogue receptor (GHS-R) [2], is actively secreted from the fornix [3–5] and has a marked effect on the promotion of food intake [6]. Ghrelin leads to increased weight gain by preventing a decrease in the ability to taste [7, 8], and also has anti-inflammatory activity [9–11]. Therefore, an important aspect of a subtotal gastrectomy that preserves the fornix is the establishment of an appropriate oncological resection line that allows the preservation of function. The transhiatal approach to the lower esophagus has been proposed by the GEJ Carcinoma Working Group in Japan as a surgical option for surgery of an adenocarcinoma of the gastroesophageal junction (GEJ) with a length of esophageal invasion of less than 4 cm, [12]. In this operation, the reconstruction method may differ depending on the location of the resection of the esophagus. Thus, accurate localization of the tumor is mandatory for performing a subtotal gastrectomy and surgery for adenocarcinoma of the GEJ.

The current major preoperative marking methods for determination of tumor location are clipping or tattooing near the tumor. The clipping method is a very useful for identifying tumor location, especially in early gastric cancer, but more visual and easier-to-understand method of capturing lesion location from the serosal side of the stomach could be considered. And the tattooing method often have a problem of spreading too far into the stomach wall to pinpoint exact tumor location. We now employ the Firefly technology of the daVinci Xi system and intraoperative upper gastrointestinal endoscopy, which allows near infrared ray-guided surgery (NIRGS) for the accurate localization of the tumor to perform subtotal gastrectomy and surgery for adenocarcinoma of the GEJ. NIRGS can pinpoint the location of tumor accurately and easily from the serosal side of the stomach, potentially leading to preoperative marking reforms. The aim of this study was to evaluate the feasibility and short-term surgical outcomes of patients with upper gastric cancer or adenocarcinoma of the GEJ who underwent NIRGS.

## Methods

### Study design

This retrospective study included patients who had been admitted for subtotal gastrectomy and surgery for adenocarcinoma of GEJ from March 2019 to December 2019 at Nagoya City University Hospital in Japan. We performed NIRGS for evaluate the accurate tumor location. NIRGS was not performed for routine distal gastrectomy, proximal gastrectomy and total gastrectomy for diseases not invading esophageal junction. In addition, no cases scheduled for subtotal gastrectomy and surgery for adenocarcinoma of GEJ were excluded from NIRGS.

### Operative technique

The daVinci Xi surgical system (Intuitive Surgical Inc., Sunnyvale, CA, USA) was used to perform robotic surgery for patients with upper gastric cancer or adenocarcinoma of the GEJ with a length of invasion of the esophagus of less than 4 cm. The length of tumor invasion of the esophagus was assessed with upper gastrointestinal endoscopy and upper gastrointestinal angiography.

**Fig. 1:**
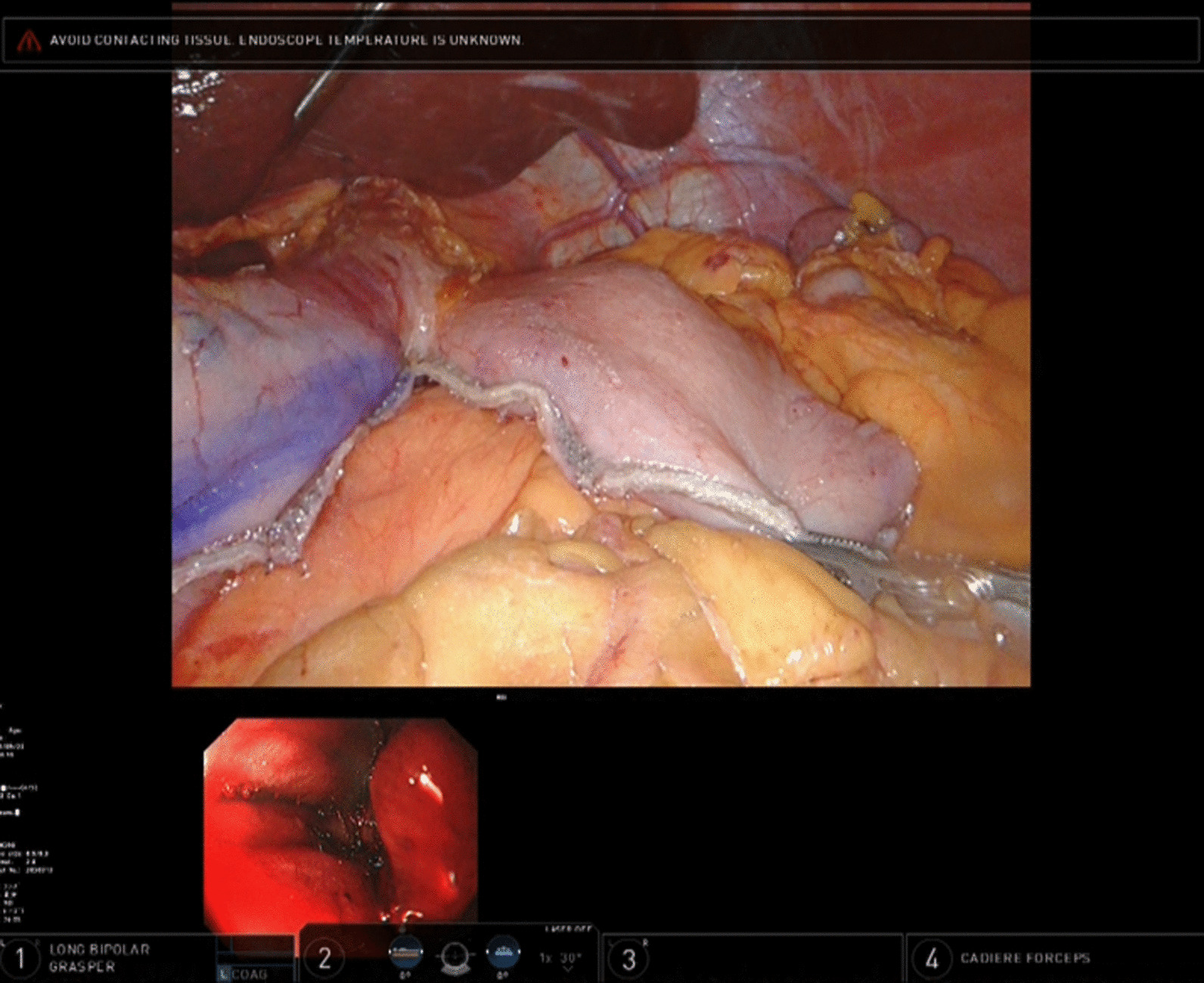
Case of upper gastric cancer. An upper gastrointestinal endoscope is inserted to the stomach, and both the surgeon and endoscopist check the location of the tumor and invasion range with Tile Pro mode on the daVinci Xi

**Fig. 2:**
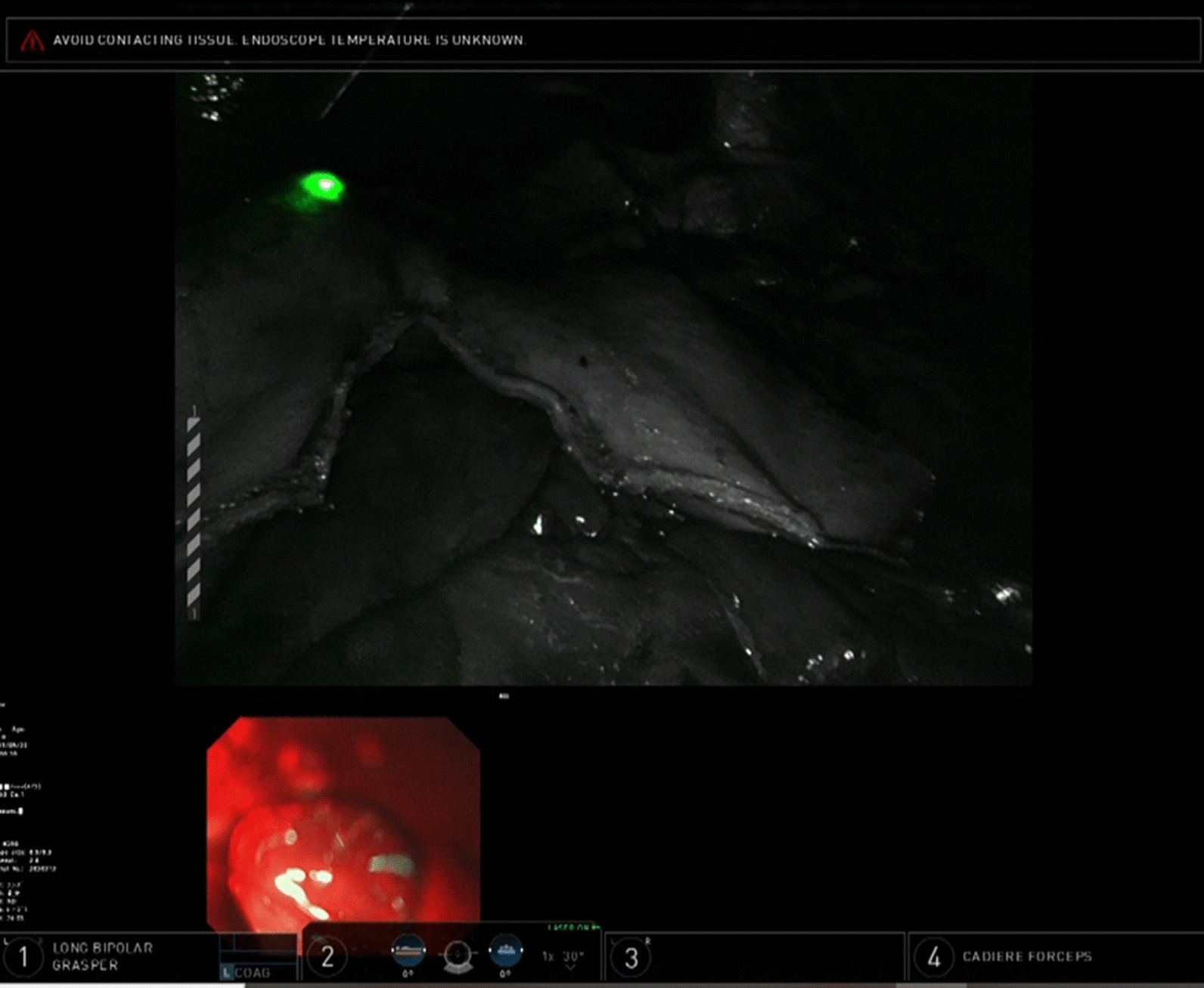
Case of adenocarcinoma of the GEJ underwent NIRGS. The surgeon can detect the near infrared ray contained within endoscopic light as green light with Tile Pro mode on the daVinci Xi

**Fig. 3:**
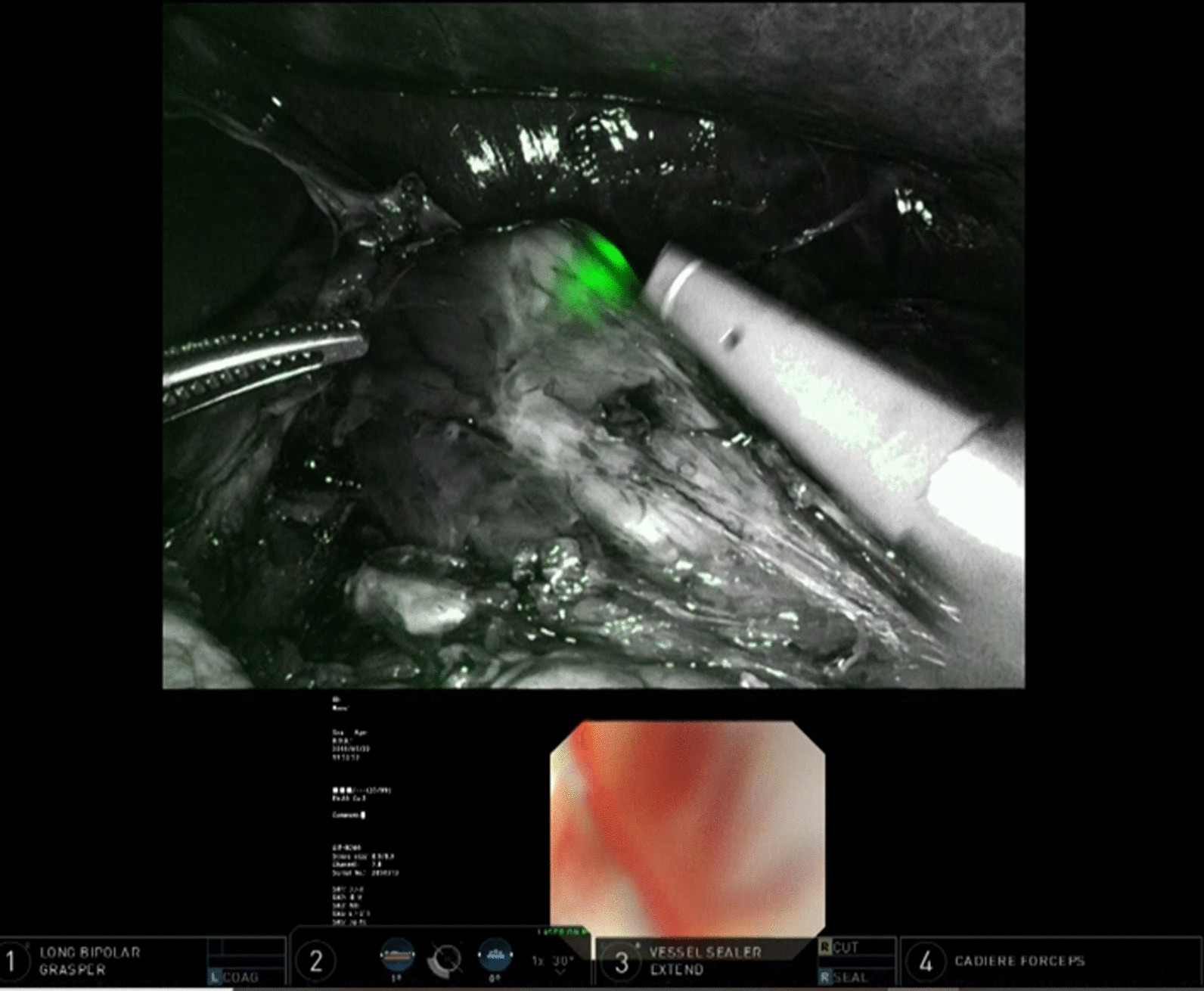
Technic of NIRGS. The tip of the endoscope was pressed to the wall of the anterior esophagus near the oral wedge of tumor

**Fig. 4:**
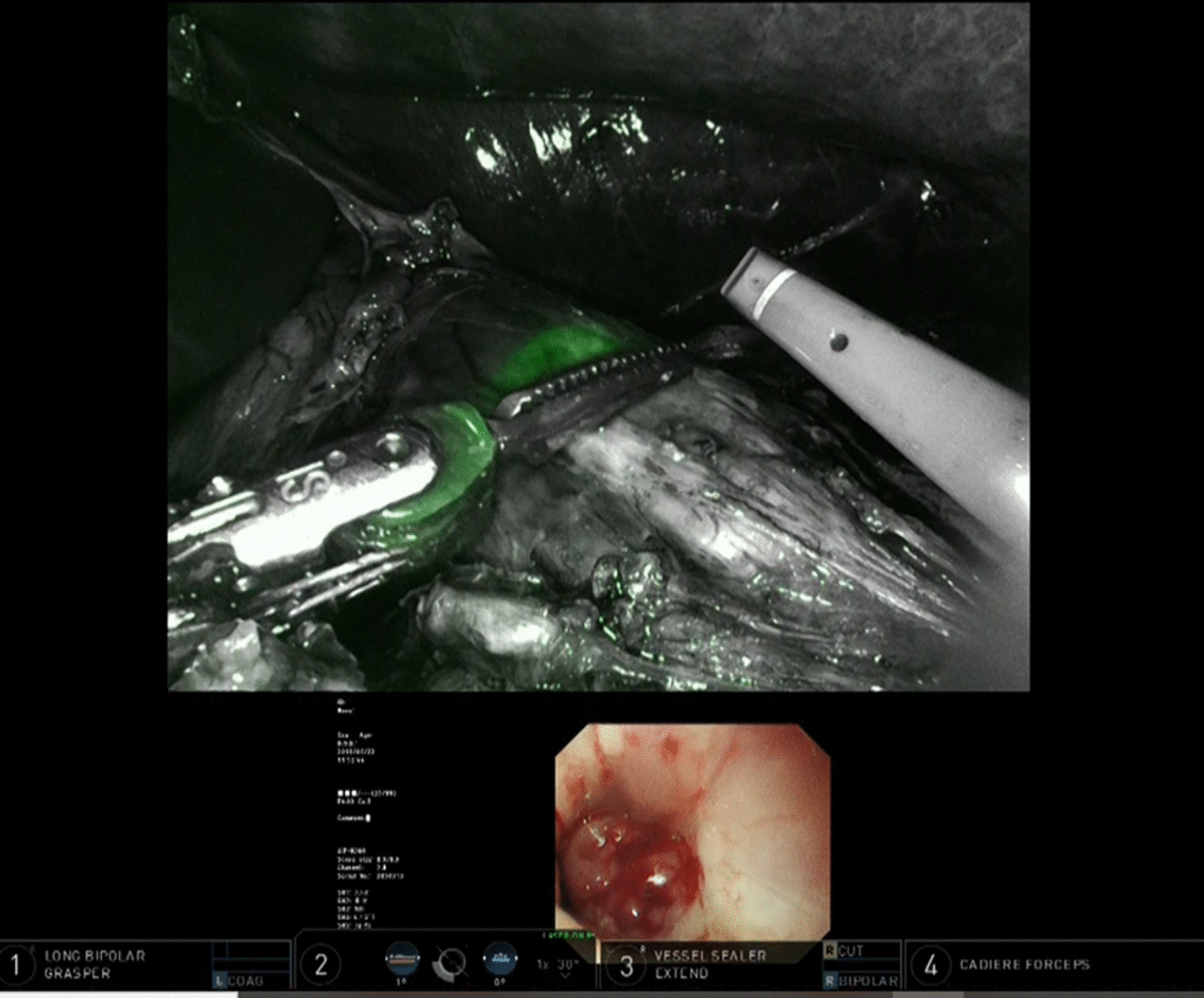
Technic of NIRGS. The line which the tip of the upper gastrointestinal endoscope is pressed to the anterior esophageal wall is recognized as surgical cut line

**Fig. 5:**
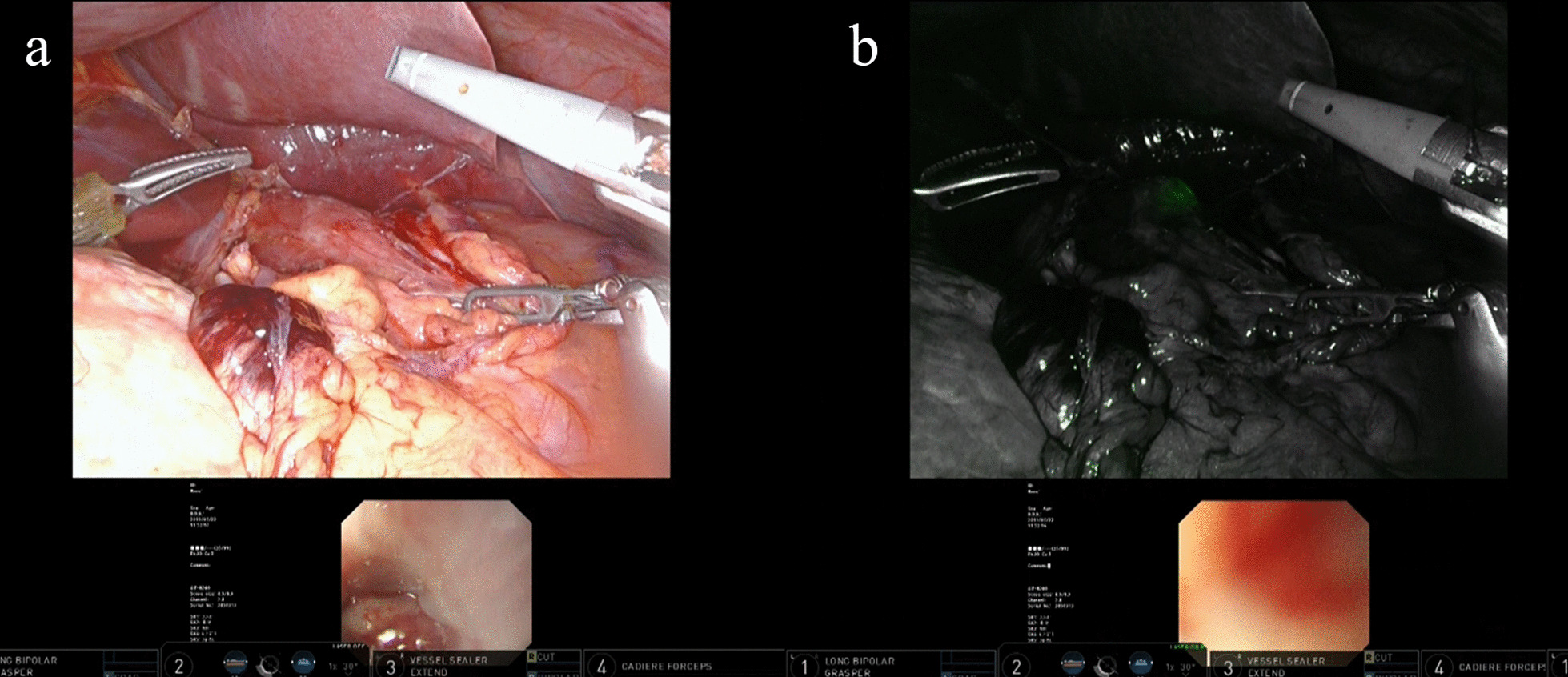
Difference of NIRGS compared to normal endoscopic light. **a** The endoscopic light through the intestinal wall in normal visible light mode cannot be seen. **b** However, the endoscopic light as a green light through the esophageal wall on the Firefly mode is clearly detected

**Fig. 6:**
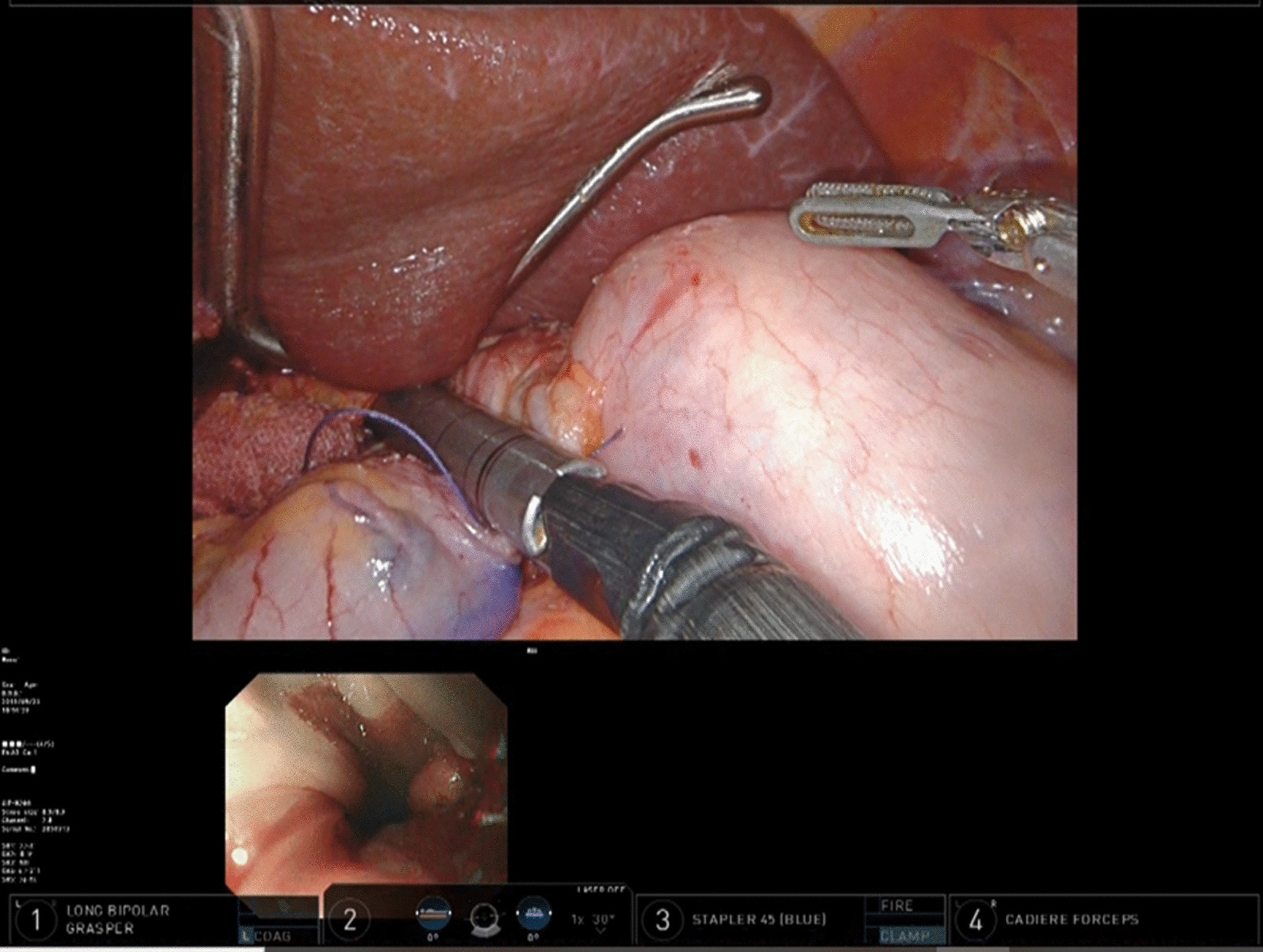
Resection of stomach. After the resection line with the margin from the tumor based on the visible endoscopic light is marked, the stomach is resected

**Fig. 7:**
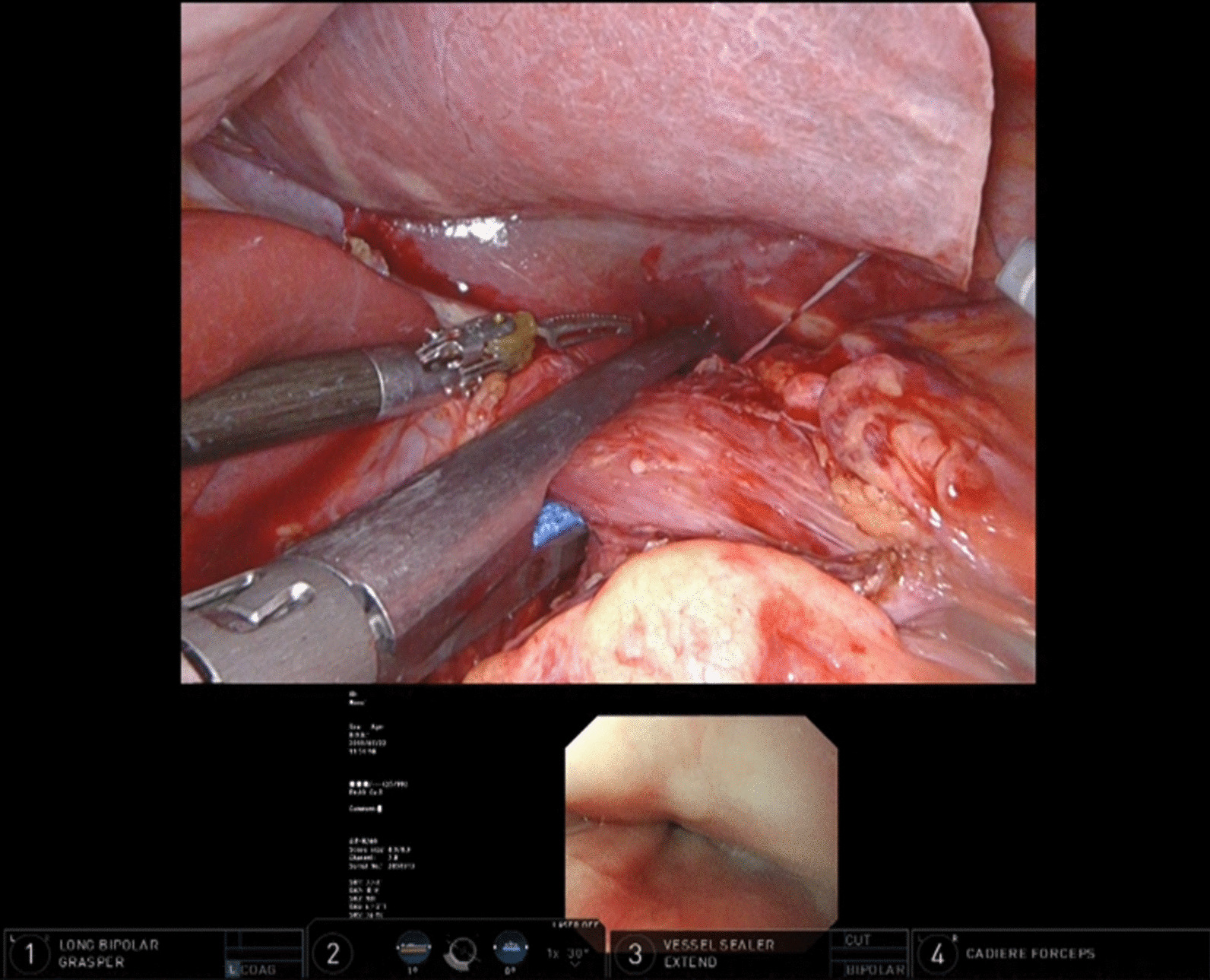
Transection of esophagus. After the resection line with the margin from the tumor based on the visible endoscopic light is marked, the esophagus is transected

After induction of general anesthesia, patients were positioned in the supine position with the left arm fixed to the trunk. Depending on the situation, the patient’s head was angled upward at an angle of 0–15 degrees. An upper gastrointestinal endoscope (Olympus Corporation, Tokyo, Japan) was inserted up to the esophagus. The lymph nodes were dissected as appropriate, and the lesion of the tumor was observed before resection of esophagus or stomach was performed. The endoscope was inserted into the stomach or lower esophagus, and both the surgeon and endoscopist checked the location of the tumor and the range of invasion with the use of the Tile Pro mode of the daVinci Xi system (Fig. 1). The Firefly mode was then activated on the daVinci Xi system. The surgeon can detect the near infrared ray contained within endoscopic light as green light (Fig. 2). The tip of the endoscope was pressed to the wall of the anterior stomach or esophagus (Fig. 3), that is surgical cut line (Fig. 4). Although the normal visible light provided by the endoscope cannot be seen through the gastric wall, the Firefly mode provides a green light that can be clearly detected through the gastric or esophageal wall (Fig. 5). Additionally, the Tile Pro mode of the daVinci Xi system allows not only the endoscopist, but also the surgeon to visualize the endoscopic light while viewing the console. A resection line that allowed an appropriate margin for the tumor was marked with the guidance of the visible light of the endoscope. Finally, the robotic EndoWrist Stapler system or SureForm Stapler system was used to transect the stomach or esophagus (Figs. 6, 7). The surgical specimen was retrieved through a small incision in the navel, and the resection margin was checked.

### Analysis

The patients’ records were retrospectively examined to tumor size, determine the extent (mm) of the tumor invasion of the esophagus, operative procedure, visualization of near infrared ray contained within endoscopic light, distance from the proximal margin from the tumor to the surgical cut line on rapid histopathology and in the permanent preparation, including the operative videos and extracted specimens, and long-term outcome including local recurrence. Based on these, we investigated the safety and efficacy of NIRGS.

## Results

Between March 2019 and December 2019, 12 patients with upper gastric cancer or adenocarcinoma of the GEJ underwent NIRGS provided by the daVinci Xi surgical system and intra-operative upper gastrointestinal endoscopy at Nagoya City University Hospital, Nagoya, Japan (Table 1). Of the 12 patients, 8 had gastric cancer, of whom 2 had tumor that had invaded the esophagus, and 4 had adenocarcinoma of the GEJ. No cases of planned but failed NIRGS were identified.

**Table 1:** Short-term surgical outcomes of NIRGS in gastric cancer and adenocarcinoma of the GEJ

Case	Disease	Tumor location	Tumor size (mm)	Tumor invasion of the esophagus (mm)	Operation	Visualization (NIRGS)	Tumor invasion in oral wedge Rapid histopathology	Histopathological proximal free margins (mm)	Reconstruction	Local recurrence, Post operative period (month)
1	Gastric cancer	U, Less	80 × 50	0	RDG	detectable	No	5	Billroth I	No, 24
2	Gastric cancer, IPMN	M, Ant	10 × 7	0	RGD + DP	Detectable	No	15	Billroth II	No, 24
3	GEJ cancer (Siewert type II)	G = E, Post	30 × 20	5	RLEPG	Detectable	No	15	Esophagogastrostomy	No, 23
4	Gastric cancer	M, Post-Less	10 × 8	0	RDG	Detectable	No	45	Billroth I	No, 23
5	Gastric cancer	UME, Post	146 × 84	5	RTG + DP	Detectable	No	16	Roux-en-Y	No, 20
6	GEJ cancer (Siewert type II)	E = G, Circ	50 × 40	20	RLEPG	Detectable	No	20	Esophagogastrostomy	No, 19
7	Gastric cancer	U, Post	35 × 43	0	RDG	Detectable	No	30	Billroth II	No, 18
8	Gastric cancer	UM, Less-Ant-Post	80 × 62	0	RDG	Detectable	No	10	Billroth II	No, 17
9	GEJ cancer (Siewert type II)	G = E, Less	30 × 25	10	RLEPG	Detectable	No	8	Esophagogastrostomy	No, 17
10	Gastric cancer	U, Less-Ant	40 × 23	0	RDG	Detectable	No	6	Billroth I	No, 16
11	Remnant gastric cancer	MU, Circ	65 × 65	5	RTG + DP	Detectable	No	7	Roux-en-Y	No, 16
12	GEJ cancer (Siewert type II)	G = E, Less	20 × 20	10	RLEPG	Detectable	No	7	Double tract	No, 15

IPMN: intraductal papillary mucinous neoplasm, GEJ: gastroesophageal junction (GEJ), U: Upper part, M: Middle part, L: Lower part, E: Esophagus G: Gastric, Less: Lesser curvature, Ant: Anterior, Post: Posterior, RDG: robotic distal gastrectomy, DP: distal pancreatectomy, RLEPG: robotic lower esophageal proximal gastrectomy, RTG: robotic total gastrectomy

In all the patients, the near infrared ray contained within endoscopic light was clearly visualized; a green light was observed with the daVinci Xi surgical system in Firefly mode. NIRGS was useful for localizing the tumor in all 12 patients. In addition, the stomach or esophagus could be resected with a reasonable margin, because the green light could be easily seen. In 3 patients with gastric cancer, Billroth I reconstruction was possible after the tumor was resected with an adequate margin.

The ability to obtain an accurate resection line with NIRGS was useful for patients with adenocarcinoma of the GEJ, and allowed the surgical options of direct anastomosis, esophagogastrostomy, or double tract reconstruction (Table 1). For all 12 patients, rapid intraoperative histopathological examinations of the resected specimens showed negative margins. None of the patients required additional resections. In addition, there was no evidence of carcinoma invasion at the proximal margins with histopathological examination in permanent specimen. No local recurrence has been observed to date in all cases (Table 1).

## Discussion

The goal of surgical treatment for upper gastric cancer in our institution is curative intent with as much gastric preservation as possible. Subtotal gastrectomy, which can preserve the fornix in gastric cancer, is thought to result in better postoperative QOL than total gastrectomy [1, 13, 14]. One of the reasons that subtotal gastrectomy is better for QOL is that ghrelin is secreted from the fornix [7, 8, 15–18]. A randomized phase III study found that a left thoracoabdominal surgical approach could not be justified for patients undergoing surgery for cancers of the cardia or subcardia, because it did not improve survival over the transhiatal abdominal approach and led to increased morbidity in patients [19]. We performed the transhiatal approach for patients with adenocarcinoma of the GEJ and less than 4 cm of tumor invasion of the esophagus. We took into consideration the fact that the location of the dissection of the esophagus affects reconstruction. We choose double-tract reconstruction for patients requiring resection high in the esophagus, because the visual field in the mediastinum is not sufficient to perform an anastomosis of the remnant esophagus to the remnant stomach. Therefore, an accurate identification of the position of the tumor is important for visualization and to separate the esophagus or stomach from the tumor with appropriate margins. NIRGS, which uses the Firefly technology of the daVinci Xi system and intraoperative upper gastrointestinal endoscopy is a new technique for providing an accurate localization of the tumor.

Robotic surgery using the daVinci Surgical System for gastric cancer was approved for national medical insurance converge in Japan in April 2018. Since then, it has been rapidly adopted. With robotic surgery, we can operate intuitively because it provides multi-articular function, stereopsis, minimizes tremors when the instruments are moved, and the function of motion scaling leads to a compact operation. In addition, the TilePro function allows the surgeon to confirm vascular construction by 3-dimensional CT angiography images or endoscopic imaging during surgery without leaving the surgeon console. However, we believe that it is necessary to understand the features and characteristics of the daVinci Xi system to make the most of its capabilities.

In our institution, before NIRGS, the preoperative marking method involved clipping near the tumor or tattooing the anterior wall of the stomach to make marks concentric with the tumor using an endoscope. In patients who received injections of ink, the tattoos often spread too far within the stomach wall, rendering accurate localization of the tumor impossible. And the difference in the location of posterior cancer and the concentric anterior wall to which the ink is applied. The clipping method as preoperative marking is indispensable as the proximal boundary, especially in early gastric cancer, and it has been possible to confirm the location and margin of the tumor from the location of the clip in the extracted specimen. However, clipping method has a risk of clips being caught in the stapler during transection of the organs in the case when the space between the marking clips and GEJ is very narrow in conduction subtotal gastrectomy. If the clips could be more accurately recognized as visual information from the serosal side of stomach, this would lead to more accurate identification of the tumor position and setting of the line of dissection. In NIRGS, the green light identified by the Firefly on daVinci Xi Surgical system is pinpointed and can be more precisely recognized from the serosal side and from a distance. NIRGS can also be easily performed at the touch of a button in the surgeon's console during surgery and has the advantage that not only the endoscopist but also the surgeon can confirm the tumor location on time from the endoscopic findings, allowing double-checking.

Takahashi et al. described the mechanisms involved in the actions of NIRGS. The white light of the endoscope contains near infrared rays and visible light. While near infrared rays can penetrate biological tissues up to 2 cm, visible light is reflected and cannot pass through the tissues. Near infrared rays that penetrate through the intestinal wall cannot be perceived when the system is in the normal visible light mode, although the near infrared ray mode of the laparoscope can detect and visualize the wavelengths of near infrared rays coming from the endoscopic light source, because clinically approved near infrared ray imaging applications that use indocyanine green (ICG) operate in the near infrared window (700–900 nm) with excitation wavelengths of 740–800 nm and emission wavelengths of 800–860 nm [20]. In spite of the thick walls of stomach, NIRGS clearly identified the green light of the endoscope in the Firefly mode that was shining through the gastric wall. An appropriate resection line determined by NIRGS results in safe reconstruction and allows preservation of an appropriate remnant stomach. However, more cases need to be collected, and long-term surgical outcomes need to be evaluated.

## Conclusions

NIRGS is very simple to perform with the use of Firefly technology provided by the daVinci Xi system and intraoperative upper gastrointestinal endoscopy that accurately points out the location of the tumor. We believe that it will be useful for reconstruction after resection of upper gastric cancer and adenocarcinoma of the GEJ. The ability to determine an accurate resection line should improve patients’ postoperative QOL.

### Limitations

In this study, the number of cases is still small, and it is considered necessary to accumulate cases in the future. In addition, the observation period is short, and it is necessary to consider the long-term prognosis.

## Data Availability

The data that support the findings of this study are available from corresponding author but restrictions apply to the availability of these data, which were used under license for the current study, and so are not publicly available. Data are however available from the authors upon reasonable request and with permission of corresponding author.

## Abbreviations

QOL: Quality of life
GHS-R: Growth hormone secretagogue receptor
GEJ: Gastroesophageal junction
NIRGS: Near infrared ray-guided surgery
VAS: Visual analog scale
